# Treatment of Anæmia by Enemata of Defibrinated Blood

**Published:** 1889-12-07

**Authors:** 


### TREATMENT OF ANÆMIA BY ENEMATA OF DEFIBRINATED BLOOD.

Dr. Antiq, in his thesis at Lyons, advocates this procedure, adducing the results of a number of cases under the care of Prof. Teissier, to whom the credit (?) of the suggestion is entirely due. They allege that the gravest chloroses, attended in some instances with frequent syncope, exhibited remarkable improvement, as did others in which the temperature was over 104deg. F. The number of the red corpuscles and the colouring matter were increased, the appetite improved, even becoming insatiable, as did the general feeling of health, while the body gained in weight, though the elimination of urea and phosphoric acid was augmented, indicating the activity of the nutritive processes. The only inconvenience was the occasional occurrence of colic, which was, however, easily relieved by a few drops of laudanum. They consider that we have in defibrinated blood a means of administering at once iron, oxygen, and the blood salts. But though facts are facts—if, indeed, they be facts, and not fancies—it is hard to reconcile these assertions with the conclusions of other and cautious observers, that blood is by no means easy of digestion, as might be inferred from its passage per anum in gastric ulcer, or when otherwise swallowed, and that though Brücke and Voit have shown that the rectum is not so incapable of digesting albumens other than peptones, as Czerny and Latschenberger maintained, its peptonising and absorbing power is very limited, so much so, indeed, that rectal alimentation, even if the rectum does not refuse to retain the enemata, can only be looked upon as a last resource to delay rather than to avert inanition, when no food can be taken by the mouth. The serum albumen and salts might be to some extent absorbed, but the red corpuscles would demand an amount of digestion of which one can scarcely believe the rectum to be capable in order to produce any palpable results.

